# Prognostic and predictive factors of immune checkpoint inhibitor therapy in urinary bladder cancer

**DOI:** 10.3389/pore.2026.1612333

**Published:** 2026-03-02

**Authors:** Melinda Váradi, Balázs Magyar, Ádám Széles, Sára Korda, Bernadett Németh, Barbara Simon, Henning Reis, Csilla Oláh, Orsolya Horváth, Bálint Dér, Péter Nyirády, Tibor Szarvas

**Affiliations:** 1 Department of Urology, Semmelweis University, Budapest, Hungary; 2 Institute of Pathology, University Medicine Essen, University of Duisburg-Essen, Essen, Germany; 3 Department of Urology, University of Duisburg-Essen, Essen, Germany; 4 Department of Genitourinary Medical Oncology and Pharmacology, National Institute of Oncology, Budapest, Hungary

**Keywords:** histological subtypes, microbiome, molecular subtypes, PD-L1, TMB

## Abstract

Immune checkpoint inhibitor (ICI) therapy has become a firmly integrated component of the systemic treatment repertoire for locally advanced and metastatic urothelial bladder cancer (UBC). Over the past decade, multiple ICIs have demonstrated meaningful clinical activity, and their indications have expanded across treatment lines, including second-line therapy after platinum, first-line therapy for cisplatin-ineligible disease, avelumab maintenance following chemotherapy, and, more recently, combination strategies such as pembrolizumab plus enfortumab vedotin. Despite these advances, patient responses to ICIs remain highly heterogeneous. While a subset of patients achieves substantial tumor regression and long-term survival, a considerable proportion derives little or no benefit. The rapidly evolving therapeutic landscape - encompassing antibody-drug conjugates, targeted agents, and perioperative ICI approvals - further emphasizes the need to identify which patients are most likely to respond to immunotherapy. Given the marked variability in therapeutic sensitivity and the increasing availability of alternative effective treatments, accurate prediction of ICI efficacy is becoming increasingly crucial for personalized treatment selection. In this review, we provide a comprehensive overview of currently established and emerging biomarkers of ICI response in UBC, including PD-L1 immunohistochemistry, serum inflammatory markers, tumor mutational burden, histology and molecular subtypes, gene expression patterns and microbiome features. We discuss their strengths, limitations, and potential translational relevance, highlighting ongoing challenges and future directions.

## Introduction

Over the past decade, immune checkpoint inhibitors (ICIs) have reshaped the therapeutic landscape of urothelial bladder cancer (UBC). For many years, platinum-based chemotherapy remained the backbone of systemic treatment, with limited durable responses. The paradigm began to shift in 2016, when the programmed death-ligand 1 (PD-L1) inhibitor atezolizumab and the programmed cell death protein 1 (PD-1) inhibitor pembrolizumab were approved for platinum-refractory metastatic UBC (mUBC), establishing ICIs as an effective second-line option [1, 2]. Subsequent evidence expanded their role into earlier treatment lines: pembrolizumab gained approval as first-line therapy for cisplatin-ineligible, PD-L1-positive metastatic disease [1]. In addition, maintenance immunotherapy emerged as a new therapeutic strategy. The JAVELIN Bladder 100 trial showed that switching to the PD-L1 inhibitor avelumab following disease control with platinum-based chemotherapy significantly prolongs overall survival (OS) [3]. More recently, the combination of pembrolizumab with the antibody-drug conjugate enfortumab vedotin demonstrated unprecedented OS benefit in the first-line metastatic setting, introducing the first platinum-free frontline standard [4].

ICIs have also advanced into earlier disease stages. In the non-metastatic muscle-invasive bladder cancer (MIBC) setting, nivolumab became the first approved adjuvant ICI drug after radical cystectomy for high-risk MIBC patients [5]. Furthermore, in the pre-operative MIBC setting, neoadjuvant intensification with the ICI durvalumab demonstrated clinical benefit in the phase III NIAGARA trial, representing the first positive pre-operative ICI trial in MIBC [6].

**FIGURE 1:**
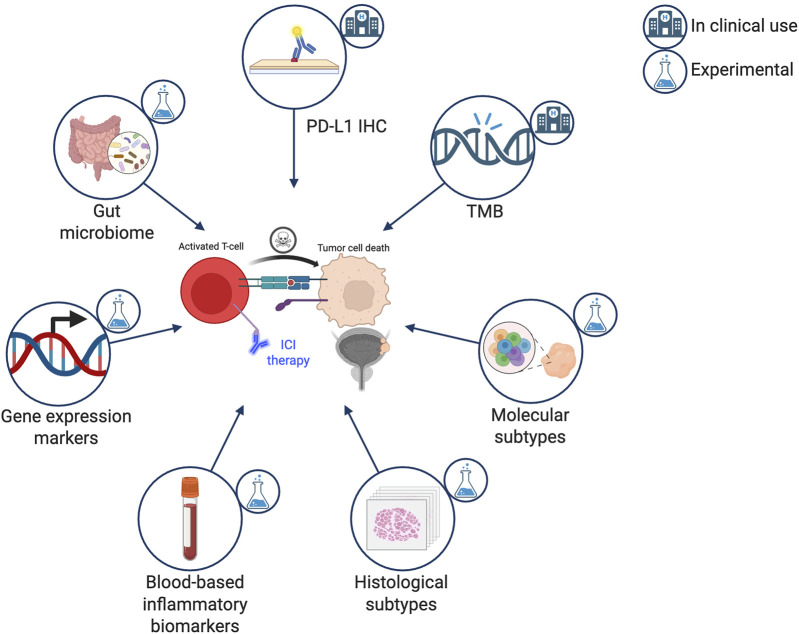
Immune checkpoint inhibitor therapy predictive biomarkers in urothelial carcinoma. ICI, immune checkpoint inhibitor; PD-L1, programmed death-ligand 1; IHC, immunohistochemistry; TMB, tumor mutational burden. Created with BioRender.com.

Despite this broad clinical integration of various ICIs in the systemic treatment of UBC, responses to ICIs remain highly variable. Only a subset of patients derives long-term benefit, while many experience primary or early resistance. Currently routinely available markers such as PD-L1 immunohistochemistry and tumor mutational burden (TMB) are limited by their accuracy and/or availability, thus highlighting a critical unmet need in UBC treatment [7]. The identification of predictive biomarkers would help guide treatment selection, optimize therapeutic sequencing, and refine combination strategies. This review synthesizes current and emerging predictive biomarkers for ICI therapy in UBC and evaluates their potential translational relevance (Figure 1).

### PD-L1 immunohistochemistry (IHC)

PD-L1 tissue expression assessed by immunohistochemistry (IHC) was the first biomarker linked to improved responses to ICIs across multiple solid tumors, and it remains one of the most extensively investigated predictive markers in mUBC. However, inconsistent findings across clinical trials highlight the limitations of using PD-L1 IHC scores as a standalone predictive marker [2, 8, 9]. According to current European Association of Urology (EAU) guidelines, PD-L1 testing is recommended only to guide the use of ICI monotherapy in patients with locally advanced or mUBC who are unfit for cisplatin-based chemotherapy. In this setting, pembrolizumab should only be used in tumors demonstrating PD-L1 expression - defined as combined positive score (CPS) ≥10 [10]. The use of different antibodies and various scoring systems may at least partly explain the limited reproducibility of PD-L1 IHC [11].

For example, in a recent study, Galsky et al. compared the results of different PD-L1 assessment approaches and identified four discordant phenotypes: PD-L1 positive by both assays, PD-L1 positive by the SP142 assay only, PD-L1 positive by the 22C3 assay only, and PD-L1 negative by both assays. UBCs that were PD-L1 positive by both assays or by SP142 alone were associated with more favorable ICI outcomes and showed increased dendritic cell infiltration. They further reported that patients with UBC exhibiting increased PD-L1 expression on immune cells experienced significantly longer OS [12]. In line with these findings, in a previous study, we compared the performance of different scoring systems and found that the immune cell score (IC) outperformed the tumor proportion score (TPS) and CPS scores for predicting ICI response - demonstrating higher specificity, sensitivity, positive predictive value and negative predictive value for radiographic response even when using the Dako 22C3 antibody, suggesting that the IC score should be prioritized when attempting to standardize PD-L1 scoring approaches [7].

PD-L1 expression is dynamic and heterogeneous, affected by multiple molecular mechanisms within both the tumor and its microenvironment [13, 14]. These biological factors, together with technical variability in PD-L1 IHC assessment, contribute to the inconsistent associations reported between PD-L1 expression and patient survival. A key limitation is that many PD-L1-negative patients still respond to ICIs. Consequently, the predictive value of PD-L1 expression could not be consistently validated in large clinical trials [15, 16].

### Tumor mutational burden

TMB, also known as tumor mutational load, denotes the number of somatic mutations present within a defined region of the tumor genome. Hypermutated tumors - characterized by high TMB - generate an increased repertoire of tumor-specific neoantigens, thereby increasing their immunogenicity [17, 18]. Because the efficacy of ICIs relies on pre-existing T-cell recognition, high TMB is associated with improved ICI response [18, 19]. Accordingly, extensive evidence shows that tumors with high TMB (TMB-H), as well as mismatch-repair deficiency (dMMR) or high microsatellite instability (MSI-H) - that serve as indicators of hypermutation - exhibit enhanced responsiveness to ICIs. These findings ultimately led to pembrolizumab’s landmark tumor-agnostic approval for dMMR/MSI-H cancers in 2017 and for TMB-H tumors in 2020, with TMB-H defined as ≥10 mutations per megabase (mut/Mb) [20, 21]. Although defects in the MMR system, MSI, and high TMB are closely related, they do not always overlap and show substantial variability across tumor types, making it essential to evaluate each metric individually [22].

Early assessments of TMB relied on whole-exome sequencing (WES), quantifying the number of nonsynonymous somatic mutations across the coding genome [18, 23]. More recently, the determination of TMB has become available through established next-generation sequencing panels, originally developed to detect actionable tumor genes, which generally cover a few megabases of genomic territory [24]. In the published UBC cohorts to date, TMB has been primarily assessed using WES, or the MSK-IMPACT (1.1 Mb) and FoundationOne CDx (F1CDx) (1.22 Mb) assays. Both panel-based assays have demonstrated their ability to predict ICI response and are approved by the U.S. Food and Drug Administration, yet they differ markedly in methodology: WES and MSK-IMPACT count only nonsynonymous coding mutations and do not exclude driver mutations, whereas F1CDx also counts synonymous mutations and indels, while excluding known drivers. Importantly, WES-derived TMB is typically reported as mutations per exome, whereas MSK-IMPACT and F1CDx report TMB uniformly as mut/Mb. These differences result in notable discrepancies in TMB values, and without a conversion formula, cross-assay comparisons remain difficult [18, 22, 25–27]. In addition to the two mentioned assays, a plethora of commercially available and validated targeted sequencing assays are available for TMB evaluation [28–30].

UBC was one of the first tumor types in which TMB was explored as a marker of ICI response. The earliest evidence came from the post-platinum mUBC cohort of the IMvigor210 trial, where higher TMB was significantly associated with a greater objective response rate (ORR) to atezolizumab [2]. These findings were subsequently validated in the same treatment setting in CheckMate 275, where higher TMB correlated not only with improved ORR (odds ratio [OR]: 2.13) but also with longer progression-free survival (PFS) (hazards ratio [HR]: 0.75) and OS (HR: 0.73) [31].

In the first-line cisplatin-ineligible cohort of IMvigor210, higher TMB characterized responders across all molecular and PD-L1 subgroups, with the highest TMB quartile demonstrating significantly prolonged survival [32]. Consistent with this, the first-line biomarker analysis of KEYNOTE-361 showed strong associations between elevated TMB and improved ORR, PFS, and OS in pembrolizumab-treated patients, and demonstrated that TMB-H patients derived benefit from either pembrolizumab monotherapy or pembrolizumab plus chemotherapy compared with chemotherapy alone, in both OS (HR: 0.69 and 0.77, respectively) and PFS (HR: 0.81 and 0.70, respectively) [33].

However, in the avelumab maintenance setting, JAVELIN Bladder 100 demonstrated no association between circulating tumor DNA-derived blood TMB and clinical or radiographic outcomes [34].

In the neoadjuvant setting, the ABACUS trial showed no association between baseline TMB and response to atezolizumab, whereas the PURE-01 trial demonstrated that higher TMB strongly predicted pathological response to pembrolizumab [35, 36].

In conclusion, evidence in UBC remains mixed, partly due to biological variability and heterogeneous TMB measurement across assays [27]. Despite its limitations, TMB remains a promising biomarker, and its predictive value improves when integrated with features such as PD-L1, MSI, immune-gene signatures, human leukocyte antigen (HLA) variation, or tumor-microenvironment metrics [24].

### Molecular subtypes

Based on their gene expression profiles, UBCs can be grouped into distinct molecular subtypes, which have also been linked to variable responses to ICI therapy. According to the consensus classification system, patients with luminal non-specified (LumNS), luminal unstable (LumU), and neuroendocrine-like (NE-like) tumors appeared to respond more favourably to atezolizumab [37]. Of the five-tiered The Cancer Genome Atlas (TCGA) classification system, the luminal-infiltrated subtype is characterized by high immune and stromal infiltration as well as elevated PD-L1 expression, and was therefore initially suggested to be sensitive to ICI [38, 39]. However, subsequent validation studies using the same classification system reported that luminal and neuronal subtypes appear to derive the greatest benefit from ICI therapy [40, 41]. In our previous work, the most favorable responses and survival outcomes were observed in TCGA-luminal infiltrated, Lund-mesenchymal, and MDA-p53-like tumors, while neuronal subtypes also showed promising ICI responsiveness [7]. In addition, an elevated neuronal signature was associated with improved response to ICI therapy even in tumors not formally classified as neuronal [7].

Beyond these conventional molecular subtype classifiers, additional ICI-specific subgrouping systems have been developed on ICI-treated UBC cohorts. One of the most comprehensive examples is provided by Hamidi et al., who profiled over 2,800 UBC specimens from four randomized atezolizumab trials using RNA sequencing, targeted DNA panels, IHC, and digital pathology. Using machine learning, they identified four transcriptional ICI-relevant subtypes - luminal desert, stromal, immune, and basal - and demonstrated that OS benefit from atezolizumab was enriched in the immune and basal groups through distinct response mechanisms [42]. Another work from Song et al. analyzed RNA sequencing data from multiple large UBC cohorts and identified four molecular subtypes, including a highly progressive, poor-prognosis group characterized by high mutation load, cell-cycle activation, and reduced transforming growth factor beta (TGFβ) signalling that appears especially suited for ICI therapy. They further confirmed in validation cohorts that patients within this subtype showed strong responses to anti-PD-L1 treatment [43].

Overall, the association between molecular subtypes and ICI responsiveness remains investigative and further validation is needed before implementing them in routine clinical use.

### Histological subtypes

Tumors of the urinary bladder consist predominantly of urothelial carcinoma (UC), which accounts for approximately 90% of all cases [44]. Of these, roughly one-third of invasive UCs show histological subtypes such as squamous, micropapillary (MPUC), plasmacytoid, glandular or other UC variations [45]. The remaining 10% of bladder cancers are heterogeneous tumor types that, according to the World Health Organization (WHO) 2022 criteria [46], are classified as pure squamous cell carcinoma (∼2–5%) [47], adenocarcinomas, sarcomas, lymphomas etc. Histological UC subtypes demonstrate different epidemiological characteristics, prognosis, and exhibit a broad spectrum of sensitivity to chemotherapy [48].

Most pivotal ICI trials in bladder cancer included tumors only if a predominant urothelial component was present. Moreover, these trials did not report outcomes according to individual histological subtypes. Because histological subtypes are diverse and single subtypes are rare, the few available retrospective or post-hoc analyses generally compared pure UC with mixed UC, without providing detailed evaluation of specific subtypes. Consequently, no robust evidence currently exists regarding the potential differential sensitivities of the various histological subtypes to ICIs.

Squamous cell carcinoma is the most frequent non-urothelial histological subtype exhibiting similarly higher TMB to UC (7 mut/Mb), almost identical levels of PD-L1 positivity with UC and less prevalent MSI-H status [49]. In contrast to the similar profile of the predictors of ICI therapy, treatment with ICIs resulted in a shorter median PFS (1.9 vs. 4.8 months, p < 0.01) and OS (9.2 vs. 20.7 months, p < 0.01) compared to pure UC, suggesting a limited ICI sensitivity of squamous cell carcinoma [50]. However, a recent large multicenter retrospective analysis did not find an OS or ORR difference between pure UCs and squamous UCs [51]. The same study found lower response rates and shorter OS for patients with an MPUC component compared to those with pure UCs, however despite the large overall cohort size of 1,511 patients, case numbers in the micropapillary group remained limited and the associations did not reach the statistical significance level [51].

In the largest retrospective case series of plasmacytoid UCs, 32% (6/19) of patients demonstrated a radiographic response, which is comparable to the response rates reported in pure UCs [52].

Similarly, the response rates reported for MPUC are comparable to those of conventional UC, with an ORR of approximately 20% in the metastatic setting with pembrolizumab [1].

In small-cell neuroendocrine carcinoma, pembrolizumab therapy has shown promising activity with all treated patients (7/7) achieving at least stable disease and 43% exhibiting an objective response in the metastatic setting [53]. Additional case series corroborated these findings, reporting high response rates to ICI therapy [54, 55].

Lymphoepithelioma-like carcinoma (LELC-B) of the bladder is a histological subtype of UC and is characterized by a syncytial growth pattern composed predominantly of large, undifferentiated tumor cells with indistinct borders and accompanied by dense lymphoid infiltration. LELC-B is frequently associated with high TMB values, enhanced PD-L1 expression and a strong immune cell infiltrate, which is characterized by CD8^+^ T cells. However, direct evidence for ICI sensitivity in LELC-B is limited; nevertheless, its molecular and histological features are strongly suggestive of high responsiveness to ICIs [56, 57].

### Blood-based inflammatory biomarkers

Blood-based inflammatory biomarkers are increasingly important in ICI-treated UBC because they provide a non-invasive, easily accessible method to explore the patient’s systemic immune status, which influences ICI response. Elevated markers such as neutrophil-to-lymphocyte ratio (NLR), or C-reactive protein (CRP) reflect protumor inflammation, as well as impaired antitumor immunity and have been consistently associated with poorer survival outcomes and reduced likelihood of response to PD-1/PD-L1 blockade [58, 59]. Inflammatory biomarkers such as NLR and CRP have emerged as promising predictors of ICI treatment in mUBC. In a recent meta-analysis of more than 6,000 patients, elevated NLR correlated with a 119% higher risk of mortality and a 90% greater risk of disease progression, underscoring its strong prognostic impact [60]. These associations remained consistent across both atezolizumab- and pembrolizumab-treated cohorts, whereas NLR showed substantially weaker predictive value in patients receiving other systemic treatments such as chemotherapy or enfortumab vedotin, highlighting its particular relevance in immunotherapy settings [50, 61]. High baseline CRP was linked to a 75% increased risk of mortality and a 58% greater risk of disease progression, emphasizing its clinical significance [60]. Moreover, incorporating dynamic CRP measurements during ICI therapy refined prognostic stratification, enabling more precise on-treatment monitoring. Patients exhibiting a “CRP flare response,” characterized by an early transient rise followed by normalization, achieved notably high response rates of 69%–75%, suggesting that CRP may function not only as a prognostic marker but also as an early indicator of ICI benefit [58, 62, 63]. Elevated platelet number before ICI therapy was also linked to worse survival rates [64]. Given the limited predictive accuracy of individual biomarkers, there is a growing need to develop integrative, multi-parameter models and composite scoring systems that more comprehensively capture the complex biological determinants of treatment response. Elevated systemic immune-inflammation index (SII), incorporating NLR and platelet-to-lymphocyte ratio (PLR), demonstrated a tendency towards worse outcomes [65, 66]. The Bellmunt risk score is a composite prognostic model for patients with UBC that incorporates Eastern Cooperative Oncology Group (ECOG) performance status, the presence of liver metastases, and hemoglobin levels [67]. Although it was initially developed for chemotherapy, it has also been shown to be associated with the prognosis of ICI-treated patients [68]. However, variability in its predictive performance led to development of the enhanced Bellmunt score, which adds further parameters such as albumin, NLR, CRP or lactate dehydrogenase (LDH) to improve risk stratification [69].

### Gene expression signature markers

Gene-expression-based (transcriptional) biomarkers play an important role in oncology, where physiological states can be highly complex and rapidly changing due to tumor evolution, treatment effects, and the development of resistance. Transcriptional biomarkers reflect real-time gene activity, and so they can capture biological dynamics that genomic profiling or proteomic approaches may miss. Another key advantage is their clinical practicality: transcriptional biomarkers can be measured efficiently and reliably using well-established platforms [70]. One of the first studies reporting a potentially ICI-predictive gene expression signature was conducted by Ayers et al., who analyzed baseline tumor RNA from pembrolizumab-treated patients. They identified and validated an 18-gene T cell-inflamed gene expression profile that was associated with clinical benefit across multiple pembrolizumab studies, including advanced UC [71]. Another study showed that in metastatic UC patients treated with nivolumab, higher epithelial-mesenchymal transition (EMT)/stroma-related gene expression in T-cell-infiltrated tumors was associated with lower response rates and shorter PFS and OS [72]. These findings could be validated in an independent real-life cohort [73]. Consistent with these results, a stromal signature was also negatively associated with treatment outcomes in pembrolizumab-treated mUBC patients in the KEYNOTE-052 cohort [74]. Li et al. developed an immune-related risk signature based on eight genes (ANXA1, IL22, IL9R, KLRK1, LRP1, NRG3, SEMA6D, and STAP2) and validated its prognostic and predictive accuracy in mUBC samples from patients who were treated with atezolizumab. The risk signature reliably separated patients with distinct survival outcomes and reflected underlying immune features. It consistently identified high- and low-risk patients, even across different tumor mutational burden groups [75]. Gene expression profiles of UBC samples receiving neoadjuvant ICI therapy (pembrolizumab) have also been examined. Necchi et al. found that immune-related gene activity generally increased after treatment, including markers of adaptive immunity, immune regulation, and innate resistance. Complete responders showed patterns suggesting pre-existing antitumor immunity, whereas non-responders displayed signs of emerging adaptive resistance with increased immune-suppressive pathways, indicating potential benefit from combination approaches [35]. Another study also in the neoadjuvant setting, applying atezolizumab, found that responding tumors showed post-treatment transcriptomic changes consistent with tumor microenvironment remodeling, including high extracellular matrix and collagen signatures. In contrast, increased proliferation and cell-cycle gene expression after treatment was linked to relapse, suggesting aggressive biology or immune escape. Tumors with stable disease showed increased immune signatures after therapy, aligning with the expected action of immunotherapy [36].

### Microbiome

The mucosal surfaces of the gastrointestinal tract are densely colonized by a diverse microbiota, the collective of organisms residing at these sites. The term microbiome technically refers to the combined genetic content of this microbiota, although the two terms are often used interchangeably. The gut-associated lymphoid tissue represents the largest immune compartment, and extensive evidence indicates that gut microbes play a pivotal role in the maturation and regulation of the immune system [76–78].

The hypothesis that host-microbiome interactions can influence ICI efficacy was first tested in melanoma cohorts in two landmark studies. These investigations not only identified microbial signatures associated with response but also demonstrated causality: experimental amplification of *Burkholderia cepacia, Bifidobacterium spp.* or specific *Bacteroides spp.* was sufficient to reinstate sensitivity to immune checkpoint blockade in otherwise non-responsive, microbiota-depleted mice [79, 80].

The importance of the intestinal microbiome in the therapeutic efficacy of ICIs is further supported by the well-established observation that antibiotic (AB) exposure - which profoundly disrupts the gut microbiome - shifts patients from responder to non-responder phenotypes. In a pooled analysis of the IMvigor210 (atezolizumab) and IMvigor211 (atezolizumab vs. chemotherapy) cohorts, patients with locally advanced or UC who received AB within ±30 days of ICI initiation exhibited significantly worse outcomes, showing reduced OS (HR: 1.44; 95% CI 1.19–1.73) and PFS (HR: 1.24; 95% CI 1.05–1.46) compared with AB-naïve patients. Notably, this effect was not observed in the chemotherapy arm [81]. Consistent findings were reported in the neoadjuvant setting by a *post hoc* analysis of the PURE-01 trial [82]. Furthermore, a current meta-analysis of 5,095 UBC patients further confirmed the negative association between AB exposure and both OS and PFS in ICI-treated patients [83]. Opioids, corticosteroids and proton pump inhibitors have also been shown to have a significant negative effect on ICI outcome in UC [84–86].

To date, relatively few studies have examined the relationship between ICI efficacy and gut microbial composition specifically in UBC. Pederzoli et al. performed sequencing on pretreatment stool samples from 42 patients with MIBC enrolled in the PURE-01 trial. Responders exhibited increased abundances of *Sutterella* and members of the *Pseudomonadota (Proteobacteria)* phylum, whereas non-responders showed enrichment of *Ruminococcus bromii* [87]. Matsumoto et al. similarly sequenced stool samples from 27 UC patients and reported that higher levels of *Veillonellaceae* were associated with worse OS and PFS (p = 0.025; p = 0.013) [88]. Benlin Wang et al. analyzed a pan-cancer ICI-treated cohort alongside a UBC cohort with healthy controls and found that ICI responders and healthy individuals exhibited higher abundances of the *Blautia* and *Parabacteroides* genera, respectively, compared to non-responders and UBC patients. Gavage of immunocompetent mice with *Blautia coccoides* and *Parabacteroides distasonis*, in combination with anti-PD-1 therapy, resulted in a significant reduction in tumor weight accompanied by increased T-cell infiltration and elevated markers of immune activation [89, 90].

Future implementation of microbiome-based interventions is advancing along three main directions: (i) fecal microbiota transplantation, (ii) modulation of the gut flora using prebiotics, probiotics, dietary approaches, and, lately, synthetic biology-engineered bacterial strains, and (iii) microbiome-derived scoring systems for patient stratification [91, 92].

In UC specifically, the ongoing phase IV trial NCT05220124 is evaluating a combined *Bifidobacterium-Lactobacillus-Enterococcus* capsule administered alongside ICI therapy [92]. Meanwhile, clinical translation is already underway, with successful fecal microbiota transplantation trials in melanoma and renal cell carcinoma, probiotic-mediated modulation using *Clostridium butyricum* (CBM588) in metastatic renal cell carcinoma, and a large-scale microbiome-based predictive scoring effort by Derosa et al. (TOPOSCORE) spanning multiple solid tumor types including UC [91, 93–96].

Contrary to the long-standing belief that urine is sterile, the bladder is now recognized to harbor its own commensal microbiota [97, 98]. Beyond the urinary microbiome, an emerging field of study is the identification of intratumoral microorganisms. Emerging evidence suggests that while these microbial communities show substantial overlap, they are not identical and display compositional differences [97, 99–101].

Although no study yet has directly correlated urinary microbiome composition with systemic ICI outcomes in UBC, urogenital microbiota profiles have been associated with tumor PD-L1 expression, and urinary dysbiosis has been linked to alterations in the tumor immune milieu [102–104]. However, in the case of the intratumoral microbiome a mechanistic link has been discovered showing *Escherichia coli*–specific adaptive immune responses dictating response to neoadjuvant pembrolizumab. In addition, *Lachnoclostridium* in the tumor microbiome associated with chemokine signatures influencing response to anti–PD-L1 therapy [105, 106].

## Discussion

Predicting response to ICI therapy in UBC remains a major clinical challenge. Although ICIs are now established across multiple disease stages, therapeutic benefit is limited to a minority of patients. This underscores the urgency of developing robust, clinically applicable biomarkers that can guide individualized treatment strategies and optimize sequencing within an increasingly complex therapeutic landscape.

PD-L1 immunohistochemistry is the most widely used ICI-predictive biomarker in clinical practice; however, its performance is modest. Variability in assays, scoring algorithms, inter- and intratumoral heterogeneity, and dynamic expression influenced by prior treatments all contribute to inconsistent results. While PD-L1 positivity is associated with higher response rates in some settings, a substantial proportion of PD-L1-negative patients also derive benefit, limiting its utility as a standalone predictor.

TMB has been explored as a surrogate for neoantigen load and immunogenicity. Elevated TMB correlates with improved outcomes in some studies, yet cut-offs lack standardization, and TMB alone does not reliably discriminate responders from non-responders in UBC.

Molecular subtyping provides a biologically grounded framework for understanding differential ICI sensitivity. Early evidence suggests that luminal and neuronal subtypes may exhibit higher responsiveness to immunotherapy; however, validation remains limited, and methodological as well as technical challenges currently restrict routine clinical application. Moreover, subtype heterogeneity and temporal plasticity further complicate reliable implementation in practice.

Histological subtypes also influence treatment responsiveness. Variants such as plasmacytoid and MPUC show variable ICI outcomes, while small-cell neuroendocrine tumors may exhibit unexpectedly high sensitivity. Larger datasets are needed to validate these observations.

Blood-based inflammatory biomarkers, including NLR, CRP, and SII, provide accessible tools that reflect host immune status. Their prognostic and predictive associations are promising but nonspecific, and standardized thresholds remain lacking.

Finally, the microbiome has emerged as a compelling modulator of immunotherapy responsiveness. While early data suggest associations between microbial diversity, specific bacterial taxa, and ICI outcomes, evidence in UBC remains limited.

Overall, remarkable progress has been made across diverse biomarker domains (Figure 1). While no single marker is sufficiently predictive on its own, integrative approaches that combine genomic, transcriptomic, inflammatory, and host-related factors are poised to significantly enhance patient stratification and further improve the therapeutic decision-making in UBC.
